# Germline testing of Iranian families suspected of Lynch syndrome: molecular characterization and current surveillance of families with pathogenic variants in *MSH2*, *MSH6*, and *PMS2*

**DOI:** 10.1097/CEJ.0000000000000916

**Published:** 2024-10-22

**Authors:** Mohammad Sina, Shiva Zarinfam, Silvia Clara Giliani, Pietro Luigi Poliani, Keivan Majidzadeh-A

**Affiliations:** a A. Nocivelli Institute for Molecular Medicine, Department of Molecular and Translational Medicine, University of Brescia, Brescia, Italy; b Genetics Department, Breast Cancer Research Center, Motamed Cancer Institute, ACECR, Tehran, Iran; c Section of Cytogenetics and Medical Genetics, Laboratory Department, ASST Spedali Civili; d Pathology Unit, Department of Molecular and Translational Medicine, University of Brescia, Brescia, Italy

**Keywords:** copy number variation analysis, DNA mismatch repair genes, genetic carrier screening, hereditary nonpolyposis, lynch syndrome surveillance, Lynch syndrome, *PMS1*

## Abstract

Lynch syndrome accounts for 3–5% of all colorectal and endometrial cancer cases, and suboptimal management of Lynch syndrome in the Middle East resulted in the underdiagnosis of mutation carriers. Probands from 24 unrelated Iranian families with a history of cancer(s) suggestive of Lynch syndrome underwent microsatellite instability analysis or immunohistochemistry, multigene panel testing, copy number variation detection, or multiplex ligation-dependent probe amplification. Pathogenic variants were identified in five patients (21%), including three in *MSH2*, one in *MSH6*, and one in *PMS2.* Microsatellite instability analysis showed the lengths of the CAT25 marker in tumor and normal samples were 149 and 148 bp, respectively. Among 21 family members with Lynch syndrome in the *MSH2* gene, identified from the three families who previously underwent cascade screening, colorectal and endometrial cancers were the most frequent. While 66% of patients had insurance that included coverage for mutation carrier screening, only one insurance provider extended coverage for next-generation sequencing. Special attention to probands and telematic management of at-risk relatives to organize blood sample collection at their convenience enhanced cascade testing 20-fold per proband. In conclusion, the age of onset and segregation analysis indicated that *PMS1* may not be a cancer susceptibility gene, and the tumor spectrum in *MSH2* pathogenic carriers is similar to Western countries. Collecting blood samples at patients’ convenience is a possible strategy to reduce the cost of identifying Lynch syndrome through cascade testing. The genetic analysis of patients for inherited cancers would optimize the current management of Lynch syndrome in Iran by omitting noncarriers from surveillance programs.

## Background

Genetic defects play a role in predisposing individuals to up to 10% of malignancies, especially breast cancer and colorectal cancer (CRC). Lynch syndrome is the most common cause of hereditary colorectal syndrome, accounting for 3–5% of all CRCs. Patients with Lynch syndrome have a pathogenic variant in one of the mismatch repair (MMR) genes, *MLH1* (OMIM# 120436), *MSH2* (OMIM# 609309), *MSH6* (OMIM# 600678), and *PMS2* (OMIM# 600259), or deletions in the *EPCAM* (OMIM# 185535). Individuals with Lynch syndrome are prone to develop hereditary colorectal and endometrial neoplasms and a wide variety of other cancers, especially at young ages (Garutti *et al*., 2023).

Mutations in short tandem repeats, also called microsatellites, typically result from DNA polymerase slippage during replication, causing strand misalignment and the insertion or deletion of repeat units. Microsatellite instability (MSI) describes variations in the length of DNA, which serve as a molecular phenotype of mutated MMR genes (Li *et al*., 2020).

The CAT25 mononucleotide marker had superior sensitivity and specificity over other markers to assess MSI status (Sánchez *et al*., 2020). In up to 90% of Lynch syndrome cases, MSI and/or negative immunohistochemistry (IHC) staining for MMR proteins were detected, which is the tumor phenotype of Lynch syndrome (Lee *et al*., 2021).

The management of Lynch syndrome in the Middle East and North African countries is based on the application of clinical criteria without knowing the carrier status of individuals. In these regions, the lack of insurance coverage is one of the obstacles to perform universal tumor testing (Sina *et al*., 2020). The use of multigene next-generation sequencing and copy number variations analysis for genetic diagnosis in cancer patients meeting clinical criteria for hereditary cancer syndromes is recommended by American College of Medical Genetics (ACMG) guidelines (Mao *et al*., 2021).

The *PMS1* gene (OMIM# 600258) is listed in a limited number of multigene panels for familial colorectal cancer type X and hereditary CRCs according to the NIH Gene Test Registry (GTR) (Rubinstein *et al*., 2012; Seifert *et al*., 2019). Already, four diagnostic panels incorporated *PMS1* into their multigene panel for hereditary CRC (Seifert *et al*., 2019). Additionally, recent studies on hereditary cancers still included the *PMS1* gene in their panels (Al-Kafaji *et al*., 2023; Manotas *et al*., 2023). The *PMS1* gene’s association with cancer, however, was contradicted in the assessment of gene panels designed for hereditary cancers. ACMG guidelines recommended caution in interpreting genetic anomalies in the *PMS1* gene as genetic susceptibilities to CRC and hereditary polyposis (Mao *et al*., 2021).

In this study, 24 Iranian patients from unrelated families with a personal or familial history of cancer(s) suggestive of Lynch syndrome underwent multigene panel testing and copy number variations analysis. The aim of this analysis was to identify cancer susceptibility variants in Iranian families. Also, the current status of surveillance programs in cancer patients, insurance coverage for genetic testing, and surveillance in Iranian families were investigated.

## Methods

### Ethical compliance

This study was performed in accordance with the principles of the Declaration of Helsinki. It was approved by the Ethics Review Committee for Medical Research of Avicenna Research Institute, ethical code: IR.ACECR.Avicenna.REC.1396.24.

### Sample collection

A group of 24 Iranian subjects with a personal or familial history of colorectal, endometrial, or ovarian cancers suggestive of Lynch syndrome (Table 1) were referred to Motamed Cancer Institute, Tehran, Iran, between September 2021 and September 2022. Identification of individuals suspected of Lynch syndrome in these extended families was performed according to current clinical criteria, including Amsterdam or revised Bethesda guidelines. Also, personal or family history of CRC, endometrial, or ovarian cancer suggestive of Lynch syndrome were considered for study. The referral was performed by gastroenterologists, gynecologists, general surgeons, and oncologists who referred patients to our clinic.

**Table 1:** Summary of the outcome of genetic testing, immunohistochemistry (IHC), and microsatellite instability (MSI) analysis in the seven families

Number of families	Gene (reference sequence)	Clinical criteria	Nucleotide change, and ACMG verdict	MSI status using CAT25	Type of IHC analysis	Location of tumor for IHC analysis	IHC analysis results of MMR proteins	MLPA	Effect in protein must be near the nucleotide change	Novel or reported
Fam. A	*PMS1* (NM_000534.5)	OC and CRC < 65 years in second-degree relative	c.2295_2296del, likely pathogenic, but not a cancer predisposition gene	NA	NA	OC	NA	NA	p.His765GlufsX19	Novel
Fam. B	*MSH2 (NM_000251.2*)	Revised Bethesda Guidelines	c.(366 + 1_367-1)_(645 + 1_646-1)del, pathogenic	NA	All MMR genes	CRC	Loss of MSH2/*MSH6*	Yes	NA	Reported(Mangold *et al*., 2005)
Fam. C	*MSH2* (NM_000251.2)	Amsterdam I	c.705dupA, pathogenic	MSI-instable	All MMR genes	CRC	Loss of MSH2/MSH6	NA	p. Asp236Argfs*20	Novel
Fam. D	*MSH2* (NM_000251.2)	Amsterdam criteria I	c.842C>G, pathogenic	NA	All MMR genes	OC	Loss of MSH2	NA	p.Ser281Ter	Reported (Moussa *et al*., 2011)
Fam. E	*MSH6* (NM_000179.3)	Family history of breast cancer and CRC	c.3226C>T, pathogenic	NA	All MMR genes	CRC	NA	NA	p.Arg1076Cys	Reported (Plaschke *et al*., 2004; Rahner *et al*., 2008)
Fam. F	*PMS2 (NM_000535.6*)	Amsterdam I	c.943C>T, pathogenic	NA	All MMR genes	CRC	Loss of PMS2	NA	(p.Arg315Ter)^a^	Reported(Sugano *et al*., 2016; Guo *et al*., 2019)

ACMG, American College of Medical Genetics; CRC, colorectal cancer; Fam, family; MLPA, multiplex ligation-dependent probe amplification; MMR, mismatch repair genes; NA, not applicable; OC, ovarian cancer.

a We were not able to identify the specific location of the deletion; deletions are based on the multiplex ligation-dependent probe amplification (MLPA) results.

All at-risk members of these families or their parents received genetic counseling and signed an informed consent form prior to genetic testing. Demographic information and other required data, including gender, ancestors, clinical history, personal and familial history of cancers/polyps, age at diagnosis, current surveillance for lynch syndrome (LS), and insurance coverage, were gathered by interviews. Detailed family trees were drawn, and cancer cases mentioned in the tumor spectrum were confirmed by the pathology reports of Lynch syndrome patients.

### Multigene panel testing, copy number variation analysis, and multiplex ligation-dependent probe amplification

After the extraction of genomic DNA from peripheral blood of all probands, HiSeq 4000 sequencer (Illumina, San Diego, California, USA) was used to sequence a set of 70 genes that were analyzed using a commercial multigene panel sequencing as follows:

*AIP, ALK, APC, ATM, AXIN2, BAP1, BARD1, BLM, BMPR1A, BRCA1, BRCA2, BRIP1, CDH1, CDK4, CDKN2A, CHEK2, DICER1, EPCAM, FANCC, FH, FLCN, GALNT12, GREM1, HOXB13, MAX, MEN1, MET, MITF, MLH1, MRE11A, MSH2, MSH6, MUTYH, NBN, NF1, NF2, NTHL1, PALB2, PHOX2B, PMS1, PMS2, POLD1, POLE, POT1, PRKAR1A, PTEN, PTCH1, RAD50, RAD51C, RAD51D, RB1, RECQL, RET, SCG5/GREM1, SDHA, SDHAF2, SDHB, SDHC, SDHD, SMAD4, SMARCA4, SMARCB1, STK11, SUFU, TMEM127, TP53, TSC1, TSC2, VHL, WT1.*

The reads in the FASTQ files were aligned to the reference genome (hg19, NCBI Build 37) using the HISAT2 aligner tool (Kim *et al*., 2019). ANNOVAR stand-alone tool was used to annotate the detected indels and single nucleotide variations, which were identified by GATK HaplotypeCaller - v4.1 (Wang *et al*., 2010). Variants with a minor allele frequency of higher than 1% (minor-allele frequency < 1%) in the 1000 Genome Project, gnomAD, and Exome Aggregation Consortium (ExAC) were filtered out. The VarSome database, Franklin by Genoox (https://franklin.genoox.com), ClinVar, MutationTaster, dbSNP, and ACMG standard guidelines were used to analyze the pathogenicity of the identified variants and variant curation (Richards *et al*., 2015; Kopanos *et al*., 2019).

Copy number variations analysis was applied to identify large deletions using ExomeDepth and CONTRA (COpy Number Targeted Resequencing Analysis) packages (Li *et al*., 2012; Plagnol *et al*., 2012). Coverages of susceptible copy number variations were checked using Integrative Genomics Viewer and SAMtools to compare with control samples (Robinson *et al*., 2011).

AnnotSV tools (http://lbgi.fr/AnnotSV/) were used to annotate and interpret the pathogenicity of the identified copy number variation variants (Geoffroy *et al*., 2018). Finally, copy number variations were confirmed by multiplex ligation-dependent probe amplification (MLPA) when found in the *MSH2* gene using SALSA MLPA Probemix P003-D1 *MLH1/MSH2* according to the manufacturer’s instructions.

### Analysis of gene panel content on clinically available hereditary colorectal cancer

An assessment of currently available genetic panels was undertaken using data from the NIH GTR. The query was conducted on the NIH GTR using the term ‘Colorectal cancer’[DISNAME] AND *PMS1*[sym], retrieving relevant information for analysis. There were 15 multigene panels in the NIH GTR that still included the *PMS1* gene using the specified term.

### Functional assay using microsatellite instability and immunohistochemistry analysis

**Fig. 1:**
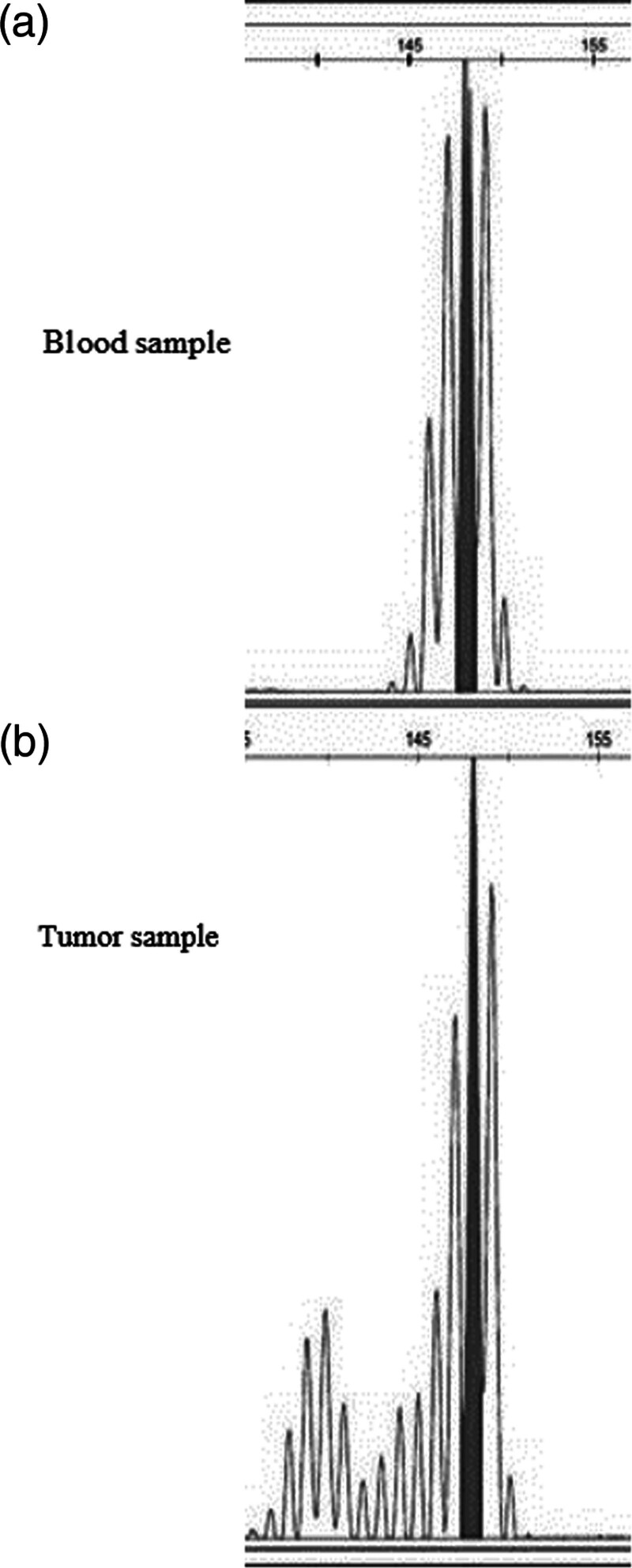
Microsatellite instability identification using PCR products and fragment analysis of CAT25 mononucleotide marker. Different ranges of the length of PCR products between blood sample (a) and colorectal cancer tissue (b) show instability. The filled peaks show the length of the fragments in each sample.

MSI analysis was exclusively conducted on CRC and blood samples of the second cousin of the proband of the family C as well as two additional healthy individuals (in three replicates) using CAT25 mononucleotide marker as described previously (Fig. 1) (Babaei *et al*., 2017). Data analysis was performed using GeneMapper 5. IHC analysis was conducted using antibodies against MMR proteins as previously described on the tumor tissue of the aunt of proband A, and all other probands (Goshayeshi *et al*., 2017).

### Cascade testing and segregation analysis

Segregation analysis was performed, and the tumor spectrum in verified mutation carriers was determined, if possible. Sanger sequencing was used to confirm the presence of pathogenic variants in the probands and their at-risk relatives. In brief, DNA was extracted from peripheral blood according to the manufacturer’s instructions (Wizard Genomic DNA Purification Kit, Promega, Madison, Wisconsin, USA). PCR was performed for the exons of interest, and then sequencing was implemented using ABI 3130 Genetic Analyzer (Applied Biosystems, Foster City, California, USA) (primers are available upon request).

Family C received personalized management and communication from specialists who coordinated the collection of blood samples from at-risk relatives within the first to fourth degree at their convenience. Offering this service to additional families faced obstacles stemming from logistical complexities or a lack of enthusiasm among other probands to cooperate.

## Results

### Mutation analysis results, segregation analysis, and immunohistochemistry

**Fig. 2:**
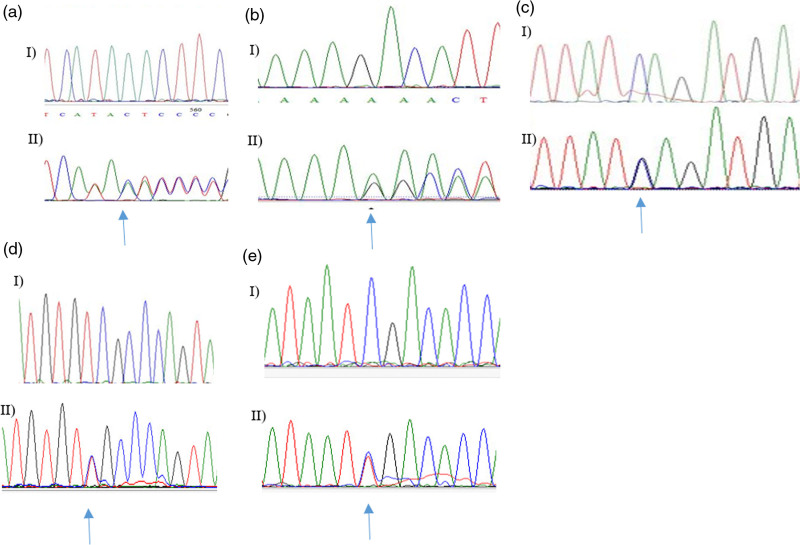
Sanger sequencing chromatogram showing DNA sequence of heterozygous pathogenic variants of six probands identified in this study, and arrows show the site of the heterozygous mutations. (I) wild type, (II) mutation carrier. (a) *PMS1* NM_000534.5:c.2294_2295 (reference sequence NM_000534.5), (b) *MSH2* (reference sequence NM_000251.2) NM_000251.2:c.705dupA, (c) NM_000251.2:c.842C>G in *MSH2* (reference sequence NM_000534.5), (d) NM_000179.3:c.3226C>T in *MSH6*, (e) NM_000535.7:c.943C>T in the *PMS2* gene (reference sequence NM_000535.6).

**Fig. 3:**
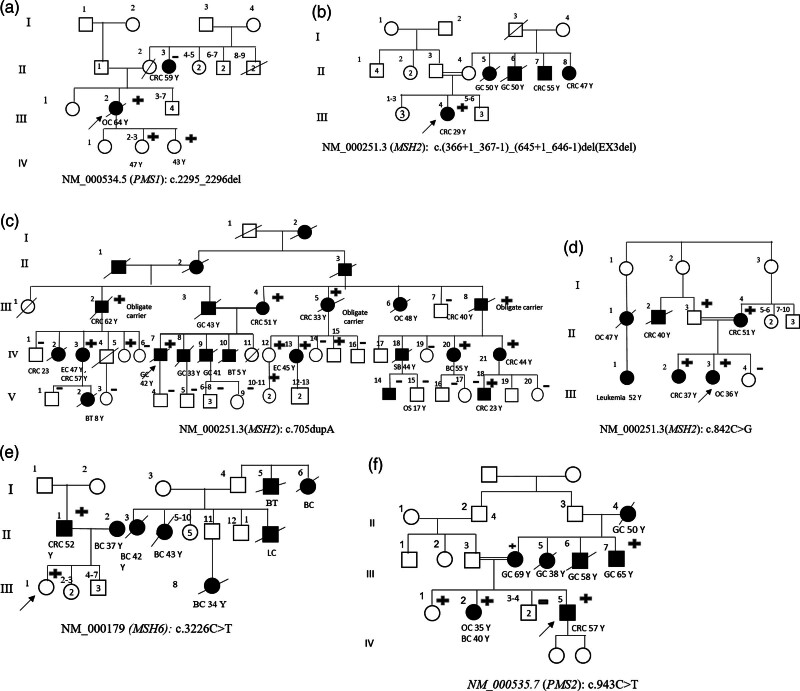
Pedigree of six families harboring heterozygote pathogenic variants related to hereditary cancers. (a) Family A, (b) family B, (c) family C, (d) family D, (e) family E, and (f) family F. Black: diagnosed with cancer, white: not affected with cancer. Probands are represented by arrows, males are shown by squares (□), females are depicted by circles (○), and spouses and siblings by horizontal lines. Generations and individuals are represented as vertical lines. In the case of consanguineous marriages (e.g. cousin marriages), a double line between spouses was drawn. Y: age at diagnosis. Cancer type is specified as follows: BC, breast cancer; BT, brain tumor; CRC, colorectal cancer; GC, gastric cancer; OC, ovarian cancer; OS, osteosarcoma; SB, small bowel cancer. Tested individuals are indicated with + if mutation carrier, - if wild type. Numbers within shapes: number of people in each generation. With the exception of obligate carriers, the cancer incidences were confirmed through pathology reports for those indicated with +. For the remaining cases, the diagnosis was established based on family history, corroborated by information provided by relatives. Numbers inside a symbol indicate the number of individuals with the same features. Numbers above a symbol indicate the number of individuals within a generation.

In this study, 24 patients underwent multigene panel sequencing, in which 18 cases did not receive a pathogenic variant diagnosis. We identified seven different variants in seven unrelated Iranian families. The clinical and tumor-related features of patients and their relatives are detailed in Table 1. Variants included one with *PMS1* gene defect and six pathogenic variants associated with Lynch syndrome as follows:

In family A (Fig. 3a), the proband (III: 2) was diagnosed with ovarian cancer at the age of 64 and was found to be a carrier of a novel frameshift pathogenic variant NM_000534.5:c.2294_2295 del in the *PMS1* gene (Fig. 2a, reference sequence NM_000534.5). The formalin-fixed paraffin-embedded was not available for further analysis. Proband’s maternal aunt (II: 3) developed CRC at the age of 59 and was found not to be a carrier of the variant. IHC analysis of MMR proteins in the aunt’s CRC tumor (II: 3) displayed intact expression, suggesting the occurrence of sporadic CRC.

In family B (Fig. 3b), the proband was a 29 years old female CRC patient (III: 4), carrier of a heterozygous exon 3 deletion NC_000002.11 (NM_000251.2):c.(366 + 1_367-1)_(645 + 1_646-1)del in the *MSH2* gene (reference sequence NM_000251.2), and four cases of LS associated cancers had occurred in her maternal family.

In family C (Fig. 3c), the proband (IV: 7) was affected with gastric cancer and met criteria for both Lynch syndrome and hereditary diffuse gastric cancer. A novel frameshift pathogenic variant NM_000251.2:c.705dupA (Fig. 2b) in the *MSH2* gene was identified in the patient, which was predicted to lead to premature truncation of the MSH2 protein. In this family, seven cases were excluded because they or their parents refused to undergo cascade testing. Thirty alive at-risk relatives, however, were analyzed for the familial mutation, and 12 alive Lynch syndrome variant carriers were identified. The remarkably high rate of cascade testing observed in this family can be attributed to facilitated sample collection. This approach led to a 20-fold increase compared to other studies and a 15-fold increase compared to six other families in this study.

This genetic anomaly was found in the proband’s mother (III: 4), who developed CRC at 51 years, and in eight additional relatives affected with Lynch syndrome-associated cancers. Notably, a maternal uncle (III: 7) and a maternal cousin (IV: 17) of the proband, were noncarriers at the ages of 72 and 50 years, without any history of cancers or polyps. This result supports the correlation of the mutation with cancer in this family. Additionally, the presence of the pathogenic variant in cancer-affected members [(IV: 21) and (V: 18)] across two generations further confirmed this link (Fig. 2c).

In this family, IHC and MSI analysis were performed on the CRC tumor sample of the second cousin (V: 18) of the proband, who was diagnosed at 23 years of age. IHC analysis showed the loss of MSH2/*MSH6* protein expression, and MSI was high (Fig. 1).

In family D (Fig. 3d), the proband was affected by ovarian cancer at the age of 36 and was a carrier of NM_000251.2:c.842C>G (Fig. 2c) in the *MSH2* gene. The loss of protein expression was confirmed by IHC in her tumor tissue. Segregation analysis revealed that both parents [(II: 3) and (II: 4)] were mutation carriers, and the mother was diagnosed with CRC at 59 years of age. The proband had two sisters; one was a carrier (III: 2) of the genetic anomaly and developed CRC at 37 years, while another (III: 4) was wild type without any history of cancer.

In family E (Fig. 3e), the presence of NM_000179.3:c.3226C>T (Fig. 2d) in the *MSH6* gene (reference sequence NM_000179.3) was confirmed in the proband’s father by Sanger sequencing, followed by IHC in her tumor tissue.

In the family F (Fig. 3f), the proband developed CRC and was a carrier of NM_000535.7:c.943C>T (Fig. 2e) in the *PMS2* gene (reference sequence NM_000535.7). One of the proband’s sisters (IV: 2) was a carrier of a genetic mutation (III: 4), developing ovarian cancer at the age of 35 and breast cancer at 40 years. The proband’s mother (III: 4) and maternal aunt (III: 7) were also carriers of the mutation and were diagnosed with gastric cancer at the ages of 69 and 65 years, respectively.

### Microsatellite instability analysis

The MSI analysis was carried out on tumor tissues using the CAT25 mononucleotide marker only in family C. The PCR product obtained from the blood sample cells of this patient was a fragment of 149 bp, while the wild-type allele was 148 bp. In the cancer specimens, there was an additional allele at 139 bp, which was absent in blood samples (Fig. 1).

### Tumoral spectrum in *MSH2* carriers

Overall, a total of 21 mutation carriers of *MSH2* pathogenic variants resulted from cascade testing of three families (18 alive mutation carriers, and three dead obligate carriers) were identified; 14 cases were cancer affected [one case with both endometrial cancer (EC) and CRC] (Table 2). Pathogenic variants in *MSH6* were confirmed in one CRC patient.

**Table 2:** Tumor spectrum and diagnosis ages in *MSH2* pathogenic variant carriers

Tumors types	Number of affected cases	Average age at diagnosis
Colorectal cancer	10	42
Gastric cancer	1	42
Endometrial cancer	2	46
Ovarian cancer	1	36
Breast cancer	1	55

The average ages at diagnosis in individuals with the confirmed (or obligate) pathogenic variants in the *MSH2* (reference sequence NM_000251.2) gene from three families.

### Insurance coverage

The participants were asked about the status of their healthcare insurance, the coverage of the costs of genetic testing, and surveillance programs. Although 28 (66%) of 42 participants had insurance that covered the costs of mutation carrier screening, one insurance provider covered the costs of next-generation sequencing. All insurance providers covered part of the cost of surveillance, including endoscopy and colonoscopy.

### Surveillance programs

Among all seven families, only 14 members of family C, including six cancers-affected and four nonmutation carriers, had participated in surveillance programs for Lynch syndrome before genetic testing. Among this extended family, six subjects, aged between 18 and 25 years, did not participate in any surveillance program. Knowing the carrier status due to genetic testing resulted in a significant optimization of Lynch syndrome surveillance in family C. Among the patients who underwent Lynch syndrome surveillance solely based on medical suspicion, 28% (4 of 14) were identified as nonmutation carriers. In addition, six nonmutation carriers between the ages of 18 and 25 years, who intended to participate in a surveillance program after the age of 25, were spared Lynch syndrome surveillance. One of the female mutation carriers informed us that she underwent a preimplantation genetic diagnosis to ensure she would have a child with the wild-type variant. The pregnancy was successful, and she gave birth to a healthy male baby.

## Conclusion

In the present study, multigene panel sequencing identified six different putative pathogenic variants responsible for Lynch syndrome, including three novel mutations.

Also, an ovarian cancer-affected patient identified with a pathogenic variant in the *PMS1* gene, is predicted to be disease causing. The median age of onset for the occurrence of ovarian cancer, however, is 63 years old in the general population (Plaxe, 2008), and the patient developed cancer at 64 years of age. Additionally, the lack of cosegregation between the *PMS1* pathogenic variants in the patient’s aunt suggests that the *PMS1* gene is unlikely to contribute to hereditary ovarian cancer predisposition. Our research further supports previous studies showing no link between the *PMS1* gene and increased cancer risk (Prolla *et al*., 1998; Landry *et al*., 2022). Consequently, the gene should not be considered in the diagnostic genetic lab as a disease-causative gene.

The National Comprehensive Cancer Network guideline recommended starting colonoscopic surveillance at the age of 20–25 or 2–5 years before the earliest CRC incidence in families with Lynch syndrome (Samir Gupta, 2022). Despite the presence of CRC at the age of 23 years in family C, some young members were recommended to start a surveillance program after 25 years of age, demonstrating that genetic and clinical counseling is a missing part of surveillance in the current Iranian healthcare system.

Cascade testing in the relatives of family C was higher than in the other families. This can be attributed to the organized blood sample collection from at-risk available relatives at their convenience. Our approach resulted in a 20-fold surge in cascade testing, a notable increase compared to the 1.5 per proband reported in other studies (Frey *et al*., 2020).

The pathogenic variant NM_000251.2:c.842C>G in the *MSH2* gene was previously reported from Tunisia (Moussa *et al*., 2011), and its role was confirmed by our study. Given the three different *MSH2* pathogenic variants characterized in our study, the tumor spectrum of confirmed *MSH2* pathogenic carriers in the present study is similar to the one described in Western countries with the highest occurrence rates of CRC and EC (Dominguez-Valentin *et al*., 2020).

Compound heterozygosity for NM_000179.3:c.3226C>T in the *MSH6* gene has been reported in the literature in three unrelated cases and two sisters with constitutional mismatch repair cancer syndrome (Okkels *et al*., 2006; Plaschke *et al*., 2006; Rahner *et al*., 2008; Jasperson *et al*., 2011). This variant is described as a rare variant with an average frequency of 0.00009 in the general population (GenomAD and ExAC) databases.

In family E, we described a CRC-affected patient (probands’ father) with the same NM_000179.3:c.3226C>T variant in the *MSH6* gene and the loss of protein expression in *MSH2/MSH6*. Pedigree analysis for the proband showed the occurrence of five breast cancers on the maternal side, indicating that further studies are required to identify the underlying gene.

Our study is in line with the previous reports confirming that germline mutation carriers of NM_000535.7:c.943C>T in the *PMS2* gene are highly susceptible to developing Lynch syndrome-associated cancers, which was confirmed by IHC (Vaughn *et al*., 2010; Borràs *et al*., 2013).

MSI analysis using CAT25 marker showed a different length of the fragment as compared with the Western population and Iran. The PCR products of the CAT25 marker with a length of <145 or >147 bp were considered the wild type in a previous study (Babaei *et al*., 2017). In this study, the fragment of the wild type was 148, which can be attributed to a different population.

Our study had several limitations, including a small sample size of only 24 cases and a limited number of genes in the panel. To identify susceptibility genes in patients with negative results, further research using whole-genome or whole-exome sequencing in trios is essential (Pope *et al*., 2021).

Strengths of this work were to demonstrate that the *PMS1* gene is probably not a predisposition cancer gene. The tumor spectrum of Lynch syndrome carriers of the *MSH2* gene is similar to what is described in Western countries with the occurrence of mostly CRC and endometrial cancers. We showed that genetic analysis of patients who meet clinical criteria for inherited cancers would optimize the current management of Lynch syndrome in Iranian patients by removing Lynch syndrome noncarriers from surveillance programs. Special consideration to probands, along with telematic managing of at-risk relatives coupled with facilitated blood sample collection resulted in a 20-fold increase in the tested relatives per proband.

## Acknowledgements

M.S.: Conceptualization, methodology, formal analysis, data curation, original draft preparation, writing – review and editing, and visualization. S.Z.: Validation, investigation, resources, data curation, ad writing – review and editing. S.C.G.: Resources, data curation, writing – review and editing, and project administration. P.L.P.: Investigation, resources, and writing – review and editing. K.M.-A.: Conceptualization, methodology, investigation, resources, data curation, original draft preparation, writing – review and editing, supervision, and project administration. All authors contributed equally.

The data that support the findings of this study are available from the corresponding author upon reasonable request. The data are not publicly available due to privacy or ethical restrictions.

### Conflicts of interest

There are no conflicts of interest.
